# “I didn’t really understand it, I just thought it’d help”: exploring the motivations, understandings and experiences of patients with advanced lung cancer participating in a non-placebo clinical IMP trial

**DOI:** 10.1186/s13063-016-1460-8

**Published:** 2016-07-20

**Authors:** Emily Harrop, Simon Noble, Michelle Edwards, Stephanie Sivell, Barbara Moore, Annmarie Nelson, on behalf of the FRAGMATIC Trial Management Group (TMG)

**Affiliations:** Marie Curie Palliative Care Research Centre, Division of Population Medicine, Cardiff University School of Medicine, 1st Floor Neuadd Meirionydd, Heath Park Way, Cardiff, CF14 4YS UK; School of Social Sciences, Cardiff University, 1-3 Museum Place, Cardiff, CF10 3BD UK; Health and Care Research Wales Support Centre, Castelbridge 4, 15-19 Cowbridge Road East, Cardiff, CF11 9AB UK

**Keywords:** Qualitative, RCT, Lung cancer, Consent, Randomisation

## Abstract

**Background:**

Few studies have explored in depth the experiences of patients with advanced cancer who are participating in clinical investigational medicinal product trials. However, integrated qualitative studies in such trials are needed to enable a broader evaluation of patient experiences in the trial, with important ethical and practical implications for the design and conduct of similar trials and treatment regimes in the future.

**Methods:**

Ten participants were recruited from the control and intervention arms of FRAGMATIC: a non-placebo trial for patients with advanced lung cancer. Participants were interviewed at up to three time points during their time in the trial. Interviews were analysed using Interpretive Phenomenological Analysis.

**Results:**

Patients were motivated to join the trial out of hope of medical benefit and altruism. Understanding of randomisation was mixed and in some cases poor, as was appreciation of trial purpose and equipoise. The trial was acceptable to and evaluated positively by most participants; participants receiving the intervention focused on the potential treatment benefits they hoped they would receive, whilst participants in the control arm found alternative reasons, such as altruism, personal fulfilment and positive attention, to commit to and perceive benefits from the trial. However, whilst experiences were generally very positive, poor understanding, limited engagement with trial information and focus on treatment benefits amongst some participants give cause for concern.

**Conclusions:**

By exploring longitudinally the psychological, emotional and cognitive domains of trial participation, we consider potential harms and benefits of participation in non-placebo trials amongst patients with advanced lung cancer and identify several implications for future research with and care for patients with advanced cancer.

**Trial registration:**

ISRCTN80812769. Registered on 8 July 2005.

## Background

Although the use of qualitative methods in trials of complex interventions in public health and health services research is well established [1], this has been far less the case in clinical trials of investigational medicinal products. For example, in a recent systematic review of the use of qualitative research in clinical trials, authors of only 5 of 296 papers (published between 2008 and 2010) were found to have reported on clinical investigational medicinal product (IMP) trials with patients with cancer [2]. Of the small amount of qualitative studies embedded in cancer IMP or surgical trials, most have been focused on exploring and finding ways of improving trial recruitment processes [3–8]. Less common have been approaches which have used qualitative methods to explore experiences of participating in the trial in terms of patient well-being and quality-of-life outcomes [9]. In trials of cancer treatments, the primary endpoint is often survival, with secondary endpoints such as quality of life measurements. Embedded qualitative studies in clinical trials allow a much more in-depth investigation of participants’ experiences and perspectives than can be captured in these kinds of structured questionnaires [9], and they are necessary for capturing the wider benefits or harms that trial participation may bring to patients, including psychological and emotional impacts. This means considering patient understandings, expectations and motivations; how patients experience the intervention itself; and what else people get out of trials if not direct medical benefits. Such findings can be used to help improve the design and delivery of future trials, treatments and services, or, as is the case in one of our current studies, they can also be presented in real time to inform trials which are on-going [10].

Research has shown how patients with cancer typically seek therapeutic benefit from research participation, although they may also be motivated by altruism [9, 11–14]. Joining a trial can also be an important means of preserving hope [11, 13, 14]. Whilst maintaining hope is clearly important for patients with cancer, there is also concern over the potential for ‘unrealistic optimism’ [13] and ‘therapeutic misconception’. This refers to situations in which patients can confuse their participation in clinical trials (which are justified on the basis of ‘equipoise’ and clinical uncertainty over best treatment options) with personalized medical care and presumed medical benefit [12, 15]. This in turn obstructs fully informed consent to participate in research [12], making patients vulnerable as research participants, at risk of making potentially poor decisions [13, 16] and likely to experience profound disappointment and loss of hope if allocated to the control arm [17, 18]. Research into how patients with cancer understand study information has similarly shown that patients focus on information about disease and treatment at the expense of information about the research, such as risks, side effects or the purpose of the trial [19]. A number of qualitative studies have also highlighted patient confusion and misunderstanding related to key trial principles such as randomisation and equipoise, along with difficulties experienced by trial staff attempting to convey this complex information [3–7, 17].

In addition to problematic communication practices, the contextualised, experiential nature of patient knowledge and decision-making has been shown to further undermine the acceptability of trial equipoise amongst patients [8, 20, 21]. Research has demonstrated how patients judge treatment outcomes subjectively on the basis of their personal circumstances and experiences; they assign their own meanings to trial outcomes and may develop preferences based on a wider or different set of concerns than those reflected in clinical outcomes [8, 20, 21]. High levels of patient trust in and expectations of their clinical team also appear to obstruct patient understanding and informed consent. Research into how participants engage with study information has demonstrated that some patients invest so much confidence in their health professionals that they see less of a need to understand study information in any detail [19], or they may perceive an urgent need to act swiftly and take decisive action, without fully engaging [22]. Studies exploring reasons for non-consent to enter trials and the acceptability of randomisation have similarly pointed to the contradiction between participant expectations for clinician direction and involvement in their treatment decisions and principles of equipoise and clinical uncertainty [3–8, 18]. Qualitative investigation is thus needed to understand why patients choose to take part in clinical trials, their understanding of important trial processes and principles, and whether their reasoning for participation can be considered to be indicative of informed consent.

Qualitative research also has a role in exploration of what effects trial participation have on patient well-being and quality of life. It is increasingly recognized that clinical trials have more complex effects than those caused by the treatment regime, and these have been investigated in research into ‘trial effects’ [23]. On one hand, trial participation might make patients feel more useful or better supported (i.e., involve psychologically mediated effects), or trial clinicians might become better informed or more careful (because they feel under observation or are required to follow a carefully researched protocol), resulting in improved outcomes. On the other hand, if patients find the consent process stressful or if it results in loss of faith in clinicians or treatments, then outcomes could be worsened [23]. Some effects may also relate to the communication and relationships between patients and professionals, changes in patients’ understanding of the meaning and cause of their symptoms, and the patient’s own activities [24]. It is argued that in these situations people often experience a wider range of changes than the more typical biomedical, psychological or quality-of-life outcomes, which suggests the significance of contextual factors as well as the process of healing [24].

The researchers in the QualFRAG study explored longitudinally the experiences of participants of a non-placebo trial called FRAGMATIC, which has recently been reported [25]. FRAGMATIC was a randomised phase III clinical trial investigating the impact of injecting daily dalteparin (a low-molecular-weight heparin) on overall survival in patients with lung cancer. Intervention and control participants were typically receiving standard chemotherapy treatment, and intervention participants were randomly allocated to also receive the daily injections. Participants were informed of their trial arm allocation immediately after randomisation had taken place, usually by one of the trial research nurses. The trial opened to recruitment in 2006 and closed in December 2011 once its recruitment target of 2200 patients was reached. The full trial protocol is available online [26].

The broad inclusion criteria and pragmatic study design of FRAGMATIC allowed for the inclusion of patients with advanced cancer; approximately 40 % of participants were expected to have advanced cancer and palliative care needs at trial entry. The advanced disease status of this patient group and the non-placebo trial design add the following more specific reasons for in-depth, qualitative investigation of patient perspectives and experiences in this and other similar studies. On the one hand, the poor prognosis of patients with advanced lung cancer might enhance their ‘vulnerability’ as research participants, their risk of distress should they not receive their preferred treatment, or their risk for other possible harms connected with the trial or intervention (e.g., daily, sustained injections). Equally, however, it is important that patients with advanced disease be given the opportunity to take part in research and that ‘gatekeeping’ based on assumptions of vulnerability does not undermine these opportunities, as has often been the case [27]. Empirical research is therefore needed which can illuminate the ‘whole-trial’ experiences of patients with different types of advanced disease who are taking part in different types of trials. As far as we are aware, no similar study has been carried out with this patient-participant group in a non-placebo clinical IMP trial.

The QualFRAG study opened during the last year of the FRAGMATIC trial. The study was aimed at exploring the experiences and perceptions of trial participants, with a particular focus on the following:

1. The psychological impact of participation in a clinical trial for patients with advanced lung cancer in both intervention and control arms, with particular emphasis on equipoise
2. The acceptability of long-term dalteparin as a therapy in advanced lung cancer (intervention arm)
3. The impact of clinical trial monitoring processes in terms of a positive attention effect and the implications for service modelling
4. How patients prioritise and manage their symptom burden over time

In this paper, we report key themes from the QualFRAG study, focusing specifically on patient motivations, understanding and experiences of participating in the FRAGMATIC trial. Implications are identified for the future design and conduct of trials with patients with advanced cancer, as well as for the routine care of such patients, including the potential use of daily dalteparin injections. Themes relating to symptoms, side effects, quality of life and patient coping will be reported separately in forthcoming publications.

## Methods

After the study received ethical approval from South East Wales Research Ethics Committee (11/WA/0118), a total of ten participants (one female, nine male) were recruited from three sites in South Wales that were participating in the trial. Eligible patients were approached by their trial research nurses between September 2011 and December 2011. Recruitment to the QualFRAG study ceased once the FRAGMATIC trial closed to recruitment in December 2011. With the exception of two patients (one of whom was never given study information due to deterioration in condition), all eligible patients at these sites entered into the study. We aimed to interview participants three times, within 18 weeks of joining the trial, 6 weeks after the first interview, and 6–8 weeks after the second interview. These time frames were chosen to maximise our pool of eligible patients in the first instance, with follow-up intervals designed to capture change whilst minimising attrition. The final number of recruited patients is shown in Table 1, as are numbers per round of interviews. The high rate of attrition, typical of palliative care research [28], was due to deterioration and in some cases death.

**Table 1:** Recruitment and attrition figures

	Interview 1	Interview 2	Interview 3	Total interviews
Control group (CG)	6	4	3	13
Intervention group (IG)	4	2	1	7
	10	6	4	20

With the exception of one patient who was interviewed in the clinic, all other patients were interviewed at home, which is considered the most appropriate setting for this patient group [29]. Several patients also had companions present during the interviews, which, given the illness status and potential vulnerability of these patients, was also considered appropriate [29]. Unfortunately, because consent was not taken from these participants, we were unable to directly use their data, although their comments provided useful context for the interpretation of patient talk. During these interviews, interviewers took steps to try to maximise patient involvement in the interview, such as turning slightly to face the patient and verification of comments made by companions [29]. Interviews were carried out by two female researchers (EH and ME) who were previously unknown to participants. They did not have clinical backgrounds, although they had some prior experience of interviewing patients or family members affected by advanced illness and have doctoral degrees in public health and health care research. A topic guide was used which covered motivations for joining the trial, understanding of trial processes and experiences with symptoms, side effects and quality of life (summarised in Table 2). The topic guide was informed by the findings of a previous sub-study [9], the clinical experience of the Principal Investigator, our study research questions and methodological preference for an interview structure which encourages participants to talk freely and develop their own stories about the trial and illness and treatment journeys more broadly. The researchers took written informed consent before the start of the first interview, which included consent to publish anonymised extracts from participant transcripts. Interviews lasted between 15 minutes and 1 h. The interviews were digitally recorded, transcribed verbatim and fully anonymised.

**Table 2:** Summary of interview topics

Interview 1 topics	Interviews 2 and 3 topics
Joining the trial • Reasons for joining the trial • Understanding of trial purpose, equipoise and different trial arms • Preferences for and responses to trial arm allocation • Understanding of and views on randomisation • Experience of receiving and accessing information on the trial Participating in the trial • IG: Experiences of daily injections (administering injections, side effects, support, continuation) • CG: Views on daily injections • Experience of attending clinics • Experience with and views on data collection processes • Perceived benefits or disadvantages of being on the trial and suggestions for improvement Treatment experiences and quality of life • Length of time receiving treatment (e.g., chemotherapy) • Understanding of treatment(s) • Responses to treatment(s) (side effects and symptom management) • Accessing information and support • Impact of illness and treatments on quality of life (daily activities, hobbies, social and family life)	Symptom burden, management and quality of life • Symptoms experienced • Symptom management and coping • Accessing support and information • Impact on quality of life • Responses to chemotherapy/other treatment • Experiences of blood clots Experiences of injecting (IG) • Administering injections and adherence • Side effects and effects on daily life • Views on continuing with and/or stopping injections Other health care and trial experiences • Experience of receiving information on treatment and illness progression • Experiences of visiting clinic and accessing health care support • Contact with research nurse and experiences of trial-related appointments and information End of study reflections (interview 3) • Views on trial arm status at time of interview (compared with at start of trial) •Perceived changes in symptoms and side effects over course of illness • Differences between expectations of and experiences with treatment • General reflections on experiences of participating in the trial and views on participating in medical research

CG, control group; IG, intervention group

The analytic framework for this qualitative sub-study was based on Interpretative Phenomenological Analysis (IPA) [30]. IPA was chosen for this study because it enables in- depth exploration of the lived experiences of participants with the aim of understanding the meaning that events has for participants based on their subjective accounts [31]. IPA is interpretative in the sense that the researcher’s conceptions and experience, as brought to the analysis, are also recognized. IPA is based on an idiographic approach beginning with a single case as a basis upon which to develop more general categories developed in a detailed case-by-case analysis. The nature of this approach means that small sample sizes are preferred, and homogeneous groups are purposively selected according to important variables [32]. In this study, our groups were defined by whether participants were control or intervention participants, with a target sample size of 6 to 10 participants per group (12 to 20 in total). Two researchers (EH and ME) analysed the transcripts for themes following this methodology. Results were verified by the research team by independent review of a selection of transcripts.

## Results

In this section, we present key themes on patient motivations for joining the trial, understanding and acceptance of randomisation, equipoise and the acceptability of intervention and control arms, engaging with trial information, and the added benefits of trials.

### Motivations for joining the trial

#### Intervention participants

Participants were asked about what motivated them to join a research trial. Participants in the intervention group expressed more egocentric motives than the control participants, as their main reason was to potentially benefit from injecting dalteparin.

*To help, to help me so I haven’t got the clots to stop the spread of the cancer… that’s what I had it for.* [I1, interview 1]

Although one participant was mindful that the treatment could benefit others in the future and saw ‘for humanity’ as a reason to participate in research, his main concern was for his own benefit.

*So I thought, well, if it prevents blood clots, you know, it’ll, for my benefit.… Obviously, for future benefit, basically selfish, for myself, you know.* [I3, interview 1]

For some participants this hope of treatment benefit seemed influenced by suggestions from a health professional that there had been some success with the injections so far. Several participants were willing to try anything that was suggested to help them, implying a degree of desperation and potential vulnerability, especially for the patient who reported having little understanding of how the treatment worked.

*Well, obviously, medically, um, they did point out that there has been some success with it…. And that was the reason that I went on to it. Anything that can help me in my present situation…, I’ll go along with*. [I4, interview 1]

*I didn’t really understand it; I just thought it’d help, you know, if they were suggesting it, it would help with the treatment*. [I2, interview 1]

Other participants in the intervention group felt obliged to try what was available to them on the grounds that it might work, rather than have nothing.

*Well, no, I’d rather have something that might prevent it rather than have nothing, you know. Half a defence is better than no defence. So, that’s what I thought, I thought, well, uh, well it mightn’t, but then again if it does.* [I3, interview 1]

#### Control participants

Most participants in the control group presented more altruistic motives for taking part in the trial. Their main reasons for taking part were to contribute to cancer research that might help other patients in the future. These participants seemed to appreciate the value of research for advancing treatments, and there was a sense that participating in research was the right thing to do. The participant below had accepted that his condition was terminal and that the treatment would not cure him, but he wanted to take part in the research to help others.

*What I’ve got is going to kill me anyway. There’s no cure. It might give me time but that’s about all…. If something can be done to help people in the future, then surely that’s got to be a worthwhile thing when it’s not taking an awful lot of time and it doesn’t take, well, it doesn’t take anything* [C3, interview 1]

Another of the control participants expressed a particular interest in participating in research and participated in more than one study. He valued the information that is generated from research, was interested in the outcomes of research and had formed a detailed understanding of how this particular treatment worked. Participating in research also provided him with a role, with activities to pass the time and help alleviate the boredom, which he experienced after having to give up work when he became ill. He found being a research participant ‘fun’ and appreciated the attention from research staff.

*Yeah, yeah, I mean, it’s something to do, you know; it’s good fun, it breaks things up. Life gets a bit boring when you are stuck like this, you know.* [C4, interview 1]

### Understanding and acceptance of randomisation

Some participants understood randomisation. Control participants spoke more on how they felt about being allocated to their trial arm, compared with intervention participants, who reported little more than feeling ‘pleased’ to be getting the injections. Most of the participants who understood the randomisation process were accepting of the outcome on the basis that it was perceived as a fair method of allocation.

*I didn’t matter to me ’cause, as the nurse said to me, they pick the names out the hat.* [C5, interview 1]

Control participants appeared more reflective and had attempted to make sense of the process and outcome of randomisation and, with the exception of one patient, were accepting of being allocated to the control arm. The exception, by contrast, was so disappointed and angered by the outcome that he withdrew from the trial shortly after we interviewed him and did not participate in any further interviews. Although he understood the principle of randomisation, he felt that his expectations had been raised and his time wasted. He considered that randomisation could have taken place before he was given all of the information to read and made to travel a distance to the hospital.

*Like I say, difficult parking and all the rest of it, I took part in so much as went along with all that was asked me and filled forms in about this, that, the next thing, uh, and I was there a couple of hours, and then they start the random thing going, um, and I wasn’t chosen, but I think if they could have pushed my name in before and I was chosen and then asked me to go down that would, I mean, well, as far as I was concerned, it was a waste of time*. [C2, interview 1]

Other patients clearly misunderstood the selection process. Several patients thought that a medical decision had been made about which arm they should be in based on some kind of clinical assessment of their condition, in one case following the results of a blood test and in the example below by somebody not known to the patient to safeguard against potential favouritism.

*Well, it’s someone who doesn’t know you doing it. So it’s not favouritism: ‘Oh yeah, that’s a friend of mine, put him on it, like so’; so, yeah, it’s cuddling up, you know. It’s just that nobody knows you; um, they make a decision on the facts that they are given, and that’s it.* [I3, interview 1]

One participant not only misunderstood how he had been allocated to the intervention arm but also seemed unsure which arm he was even in, despite the ‘unblinded’, non-placebo design of the trial. Although he was receiving the injections, he was unsure whether these contained the ‘real stuff’ as opposed to a placebo treatment.

*Well, I didn’t understand if it’s a trial…. Am I on it? Or am I not on it? Is it the real stuff I am taking? Or is it not the real stuff?* [I2, interview 1]

### Equipoise and acceptability of intervention arm

Most intervention participants understood that they were taking part in a trial but seemed to focus more on potential treatment benefits than on the nature of the trial being to investigate a medicinal product. As such, there was sometimes little or no understanding or consideration of the unknown and potentially equal risks and benefits of participating in the trial, and the principle of equipoise. For example, some participants felt that they should try all available treatment and that ‘something was better than nothing’. This motivation may have influenced a cognitive bias towards the potential benefit of treatment, which seemed to dominate much of the talk about the trial amongst intervention participants, several of whom emphasised potential anti-cancer, curative effects of the treatment.

*But apparently it could help shrink the cancers as well…. That’s what they are hoping for.* [I3, interview 1]

The focus on treatment benefit was similarly evident in the ways in which intervention participants felt about their injection regime. Although some participants did not like injections, and most participants experienced mild discomfort and pain as well as bruising around the site of the injection, they tolerated daily injections because of the potential benefits that they might get from treatment.

*I must be a bit of a masochist really, ’cause I hate needles.*… *Because normally, even on television, you see a medical programme and you see them injecting…. I look away…. Yeah, and then I got to do it myself. It’s for a purpose*. [I3, interview 1]

A couple of patients found injecting a disadvantage of being in the trial and expressed a preference to take a pill if one were available, but again were prepared to continue with the regime.

*No, you know, I wish I could take a pill rather than inject.… That’s the only thing. But obviously that’s not probably practical…. So, as I say, it is as it is, and, uh, I go along with it.* [I4, interview 1]

In the later interviews, participants continued to tolerate injecting, despite continuing discomfort, on the grounds that it is ‘doing me good’.

*I: Um, and how do you sort of feel about carrying on injecting for the rest of the trial?*

*P: Alright, yeah.*

*I: You feel happy doing that?*

*P: As long as it’s doing me good, I don’t care.* [I1, interview 2]

For intervention patients, therefore, the perceived benefits of the treatment seemed to outweigh the negative side effects (bruising, pain and discomfort).

### Equipoise and acceptability of control arm

Amongst control participants, there was also little appreciation of clinical equipoise, with the exception of the one patient (C4) who was well informed about the trial. This patient was ambivalent about being allocated to the control arm or, to use his own words, felt ‘ambidextrous’. This ambivalence seemed to reflect an understanding that it is not known whether the injections would give him a better outcome, and an appreciation of the principle of clinical equipoise.

*So, it was all straightforward, and as I said on reflection at the time, I thought, well, maybe it’s a good thing, bad thing, as I say…. I was, was I bit ambidextrous at that point, you know, because we didn’t really know whether it was sort of good, bad, indifferent or what, you know.* [C4, interview 1]

This participant had carried out his own Internet-based research on the treatment and seemed to overestimate the risks of injecting by thinking that his risk of cancer spreading may have been increased by the drug.

*The thing with the FRAGMIN is that, uh, the basic idea was if you thin the blood up a bit, then the chemo itself can access the tumour a lot easier. Uh, the flip side of that, of course, is that if you thin the blood up the chemo cells, uh, the tumour cells can also travel around a bit easier. So it’s, it’s a doubled-edged thing, is’n it?* [C4, interview 1]

Some control participants were able also to see a positive side to the control arm: that they would not have to administer daily injections. Although these participants were prepared to go into the intervention arm and recognized the potential benefits of the injections, they were also relieved that they did not have to inject, which may have helped them accept their allocation. Whilst not necessarily suggestive of clinical equipoise, by recognizing the day-to-day negatives of the intervention arm, they were able to balance out and accept their position.

*So, like I say, I didn’t mind, don’t fancy having the needle stuck in me every day.* [C6, interview 1]

Only one patient dropped out of the trial as a response to being randomised, following his allocation to the control arm (C2). For this participant, there was a different set of perceived costs and benefits associated with participation. First, unlike the other control participants, he displayed little appreciation of the value of medical trials and hence perceived few benefits from either a personal or wider public perspective. He had also had some less than positive experiences in his medical care and thus seemed less bound by the feelings of reciprocity, dependency or trust expressed by other participants.

*The things that have annoyed me so far is you go to the [name of principal hospital in city 3], you’ve got to be there for eight o’clock in the morning, they got no bed for you. So they can’t do anything until they have found a bed for you, and I’ve been there from eight until half past one, um, just hanging around.* [C2, interview 1]

This patient also understood there to be greater costs to trial participation. The patient had recently learned that he could be treated with surgery instead of chemotherapy, which meant that he would need to make additional non-treatment-related visits to the hospital to fulfil his role as a trial participant. Adding to this, the patient also lived a considerable distance from the hospital, and it seems he incurred considerable expense and inconvenience every time he attended an appointment, some of which he also felt to have been unnecessary or badly managed.

### Engaging with trial information

Many participants did not fully engage with the trial information prior to consenting to take part, no doubt explaining some of the limited understanding described above. Trial information was given at a time when patients had a lot of information to take in about their diagnosis and treatment. One participant felt overloaded with information from a number of different health professionals at a difficult and emotional time. Information about the trial added to both his information and stress burden.

*It was almost at the time I was just starting chemo, so I had a load of information from the lung nurse, from the doctor… the specialist, uh, which quite honestly was almost an overload. Then I had this trial, which is another load of information, and it’s quite a bit of an overload when your mind is in [turmoil anyway].* [C3, interview 1]

This patient’s response to ‘information overload’ was thus to ‘switch off’ or disengage. He was cautious about focusing on ‘bad things’ if exposed to too much information; his preference was for balanced information from a health professional.

*You can have too much information, and then you sit down and you only hear the bad things. But if you get the balance, as I call it, if they give you the balance, I don’t think there’s any, you need anything else. You don’t need to know the terminology and all that, because when you look it up on the Internet, it’s frightening.* [C3, interview 1]

Most participants in the intervention group also chose not to engage in any depth with the written information about the trial, preferring only to be informed of the ‘basics’. This participant reported not to have read the trial information at all, relying only on the information given by the research nurse. His focus was on simply trying something that ‘might help’:

- *P: Yeah, I got all the information sheets, but reading them [laughs]….*
- *I: So you didn’t read them, you just went with what they said, basically.*
- *P: Yeah, if they are offering you something that they say might help, you have got to try everything you know. [I2, interview 1]*

### Added benefits of trial participation

In later interviews, several patients reflected on some of the benefits that they had experienced from taking part. As described above, many patients expressed altruistic motives for participation, and one of these described what could be considered a ‘feel-good’ factor which he gained from participation, particularly given the minimal commitment needed.

*No trial ever has a negative, negativity. It always has a positive; there’s always something positive that comes out of it, even if it is only to say, ‘We don’t want to go up that route’. So, so I have no problems. I mean, I think I’m on three or four trials at the moment, yeah.* [C3, interview 3]

Some patients also perceived benefits from the additional contact trials gave with medical staff, as well as the personal qualities of these staff.

*They’ve all been interesting. I’ve met some lovely, interesting people.… If they came to me for any other trials, I would partake.* [C3, interview 3]

*Well, I think being part of the trial, you’re looked at better than if I wasn’t on the trial. You know, you’re being watched more, you know, and so, and because you see the research nurse. Otherwise, you are living on your own and you never see anyone. At least they are keep[ing] tabs on you.* [C5, interview 3]

The importance of patients’ relationships and interactions with their health professionals was similarly highlighted in the favourable descriptions participants gave of the friendly clinic environment.

*It’s laid-back; it’s, it’s like home from home. It’s, there’s no ‘oh you’re the patient, we’re the experts’.* [I3, interview 1]

## Discussion

The reported findings derived from this qualitative sub-study complement the results of the main FRAGMATIC trial and contribute to the wider literature on clinical trials with patients with advanced cancer. By exploring longitudinally the psychological, emotional and cognitive domains of trial participation, this paper provides a broader assessment of potential harms and benefits of participation in non-placebo trials amongst patients with advanced lung cancer, with important ethical and practical implications for the design and conduct of similar trials in the future.

This research enabled an in-depth exploration of what it meant to patients to be participating in the trial. This is important, as there may be negative psychological and emotional consequences of allocation to the control arm in non-placebo trials, such as disappointment and hopelessness. Equally, however, patients may experience positive psychological outcomes of taking part. Patients in this study gave a number of reasons for deciding to join the FRAGMATIC trial, which included hope of medical benefit, trying everything, interest in fulfilling a role as a research participant and altruism. The most dominant of these, however, seemed to be altruism amongst control participants and hope of medical benefit amongst intervention participants. Previous research has identified the motivating role of both of these types of beliefs, although most suggests hope of benefit to be primary [9, 11, 13, 14, 19].

At one level, our findings could be interpreted to mean that different types of people have different reasons for joining trials. However, the fact that the difference occurs between our two groups suggests that the allocation process and outcomes are likely factors. Participants were not interviewed until after they had been randomized, which would have influenced how they accounted for their status as control or intervention participants. Control participants may also have experienced more treatment-oriented motives upon consenting to be randomized, but following randomisation necessarily engaged in a process of rationalisation [17] as they reappraised and considered a wider range of reasons for participating than had they been randomized to receive the extra treatment. Following this, there was more explicit discussion and reflexivity amongst control participants on how they reacted to being allocated to the control group, as they attempted to make sense of the process and outcome of randomisation.

In this study, it might therefore have been the process of allocation to control which brought out altruistic and other secondary motives amongst control participants, who needed to find alternative rationales for participation to enable acceptance of the trial and reduce feelings of disappointment. As highlighted elsewhere, a perception of randomisation as a ‘fair’ method of allocation supported acceptance of the control arm, while an appreciation of the value of clinical trials enabled patients to experience their participation as meaningful [33]. Therefore, although altruistic expressions featured more strongly in the talk of our participants than in other studies on the motivations or attitudes of patients with cancer [11, 13, 19], this could be explained by the timing of our post-randomisation interviews, against the pre-trial timing of interviews in these other studies. In this respect, these findings could support previous observations that altruism has a secondary motivating role to trial participation, behind hope of medical benefit [13], but adds to this the observation that in non-placebo trials altruism may become primary amongst control participants once randomisation has taken place.

In terms of weighing harms and benefits, these findings suggest that intervention participants found daily injections tolerable (experiencing relatively minor physical side effects) and derived hope from their allocation outcome. Control participants (although likely disappointed at first) were also able to find alternative rationales or benefits for taking part, which helped them to accept and feel positively about their status in the trial. Whilst non-placebo trials in this population group will always be contentious, the nature of the intervention arm (daily injecting) meant that this design was perhaps easier to accept for control patients than had the intervention been less ‘invasive’. The fact that the intervention arm was a ‘supplementary’ treatment (given on top of the courses of chemotherapy given to all patients) may also have increased the acceptability of the trial for control participants. Some patients also described additional benefits of trial participation in terms of ‘feeling good’ from helping others and perceived additional contact with supportive clinical staff. Many were highly appreciative of the friendly clinic environment, which seemed to exceed their expectations, helped them to feel valued as individuals and in some cases to ‘look forward’ to their time at clinic. This adds to the literature on ‘trial effects’ by helping to explain how trials can have effects which go beyond those of the treatment, for reasons such as patients ‘feeling useful’, better supported and developing positive relationships [23, 24].

However, whilst these findings suggest psychological, emotional and social benefits for trial participants, when we consider ‘cognitive’ domains, some negative issues are identified. As in previous studies, a number of participants had misunderstood the process of random selection [3–7] and in some cases perceived treatment allocation based on clinical assessment [17]. Amongst the majority of patients, there was also little consideration of the unknown and potentially equal risks and benefits of participating in the trial [17]. This was underpinned by a view that ‘something was better than nothing’ and a possible cognitive bias towards the potential benefit of treatment, which in some cases appears to have been influenced by comments made by clinical staff. These beliefs provided the basis for intervention participants’ acceptance and adherence to their injection regime and could be considered indicative of ‘therapeutic misconception’ [12]. This raises ethical questions over the extent of ‘informed’ consent and the potential vulnerability of these patients as research participants [11, 13, 16]. It also clearly raises questions over how study information was presented to participants (including whether participant understanding was checked at the time of consent) and how it might be improved for future trials, as has been accomplished in other cancer trials with embedded qualitative designs [3, 7].

What these examples also demonstrate, however, is the importance of contextualizing patient knowledge, (mis)understandings and related decision-making [8, 20, 21, 34]. Studies of ‘non-consenters’ into trials have demonstrated reasonable understanding of randomisation but violated expectations of direction and input from their clinicians as factors undermining trial recruitment [3–8]. In this study, however, it seems that some patients misunderstood random selection and trial equipoise because of the expectations that they had of their physicians to use their expertise to make treatment decisions that were personal to them, and had entered the trial on that basis. This belief was similarly apparent in how patients approached the study information, with many describing feeling ‘overloaded’ and a preference to instead take direction from their trusted health professionals [19, 34, 35, 36, 37]. This suggests the need to go beyond overly cognitive or ‘rational choice’ models of informed consent, which assume a process of calculated assessment of medical and/or scientific information, and give due consideration to the influences of relationships of trust and dependency and the cultural narratives which incline people towards staying positive, trying everything and trusting in the ‘experts’ [13, 38].

### Limitations and implications for further research

The main limitation of this study was the lower than intended number of participants recruited, which was due to the closure of the FRAGMATIC trial, and the high rate of attrition common to research involving this patient group. The amount and quality of data collected in the interviews, and the in-depth approach to analysis using IPA, meant that in most parts the research still generated strong results, although several notes of caution need to be issued.

First, we captured only the range of experiences of those well enough to speak with us at follow-up interviews. The high rate of attrition due to deterioration and death suggests that we may have missed at follow-up those patients who experienced the most severe decline in their health, intensification of symptoms and more negative experiences. The views and experiences reported here are also nearly all those of male participants, meaning that it is not possible to draw conclusions about female experiences or to explore any differences between our male and female participants. The low number of intervention participants similarly limits the conclusions that can be drawn about patients’ experiences of injecting dalteparin over time. Out of an initial sample of four, two withdrew after the first interview and only one participant completed all three interviews. If possible, research involving further interviews with patients receiving this treatment is recommended. The extremely low entry of female participants into the study and the smaller number of intervention participants was determined by the characteristics of participants entering the FRAGMATIC trial at our three sites in this period (only one person declined to participate in the sub-study). Had the qualitative study opened earlier and at more sites, higher numbers of participants could have been recruited, and we might have been able to equal obtain equal or closer to closer to equal numbers of male/female and control/intervention participants.

A further limitation in our study design was that we were unable to interview patients prior to randomisation. As already discussed, our data demonstrated clear differences between intervention and control participants in terms of their reasons for taking part in the trial and degree of reflection on the trial, which we suggest might be explained in terms of a post-randomisation process of rationalization amongst control participants. Future longitudinal studies should aim to interview patients prior to randomisation, as this would enable a more rigorous investigation of how attitudes shift pre- and post-treatment allocation. Given our findings on patient misunderstandings and clinician influences on patient decision-making, it would also be helpful in future research to analyse patient-professional interactions and the information-giving approaches of trial staff in trial-related consultations.

Finally, it is worth noting that many of these limiting factors could have been addressed if the qualitative study had been incorporated into the main trial from its inception, as in some of our other studies. In these ‘embedded’ studies, investigation of patient experience is included as a stated secondary outcome in the main trial, and the qualitative researchers also are members of the Trial Management Group. This helps to give greater status to the qualitative work and, where appropriate, also enables changes to the trial to be recommended on the basis of these insights [8, 10, 21]. A recently conducted synthesis of findings from this portfolio of work is to be published shortly and will help to further unravel and affirm the trial experiences of participants with cancer and other advanced illnesses.

## Conclusions

Participation in the FRAGMATIC trial was viewed positively by nearly all participants, with apparent psychological, emotional and social benefits reported in the control and intervention groups. One implication of these results, therefore, is that clinical trials should be offered to patients with advanced cancer and that non-placebo designs, whilst not ideal, can be acceptable to participants who are able to find reasons other than hope of medical benefit for participating in trials, mainly altruism. However, the generally poor levels of understanding of trial purpose and processes for some patients, combined with an apparent tendency towards therapeutic misconception and limited engagement with trial information, suggest a need to improve information-giving and consent processes.

Trial staff need to recognize and try to counter the influence that they ‘naturally’ have on patient decisions [19], and likewise the expectations that patients have for treatment decisions which are determined by expert assessment as opposed to the ‘chance’ inherent in random allocation. Finding ways of better explaining clinical uncertainty, principles of random selection and laying emphasis on the fact that clinical tests and/or assessments are not used to determine treatment allocation could help [7]. Delivering such messages more informally, at times which are meaningful for patients, and without overloading patients with written information might also be steps in the right direction. Checking patient understanding and motivations at the time of consent and later intervals could also help improve how ‘informed’ patient consent in trials really is [9]. Increased public awareness of the general purpose and process of trial participation could also help to improve patient understanding in the longer term [4] whilst also addressing problems of information overload.

In terms of implications for patient care in clinical practice, daily injections of dalteparin were acceptable to our participants because of the perceived potential benefits. Whilst no survival benefit was observed in the dalteparin arm, there was significant reduction in the risk of venous thromboembolism and no difference in major bleeding events [25]. As such, this research may support the use of dalteparin in clinical practice with patients with advanced lung cancer, although further interviews would definitely be needed to confirm this, given our low number of intervention group participants. Our research also highlighted the salience of good communication, friendly clinic environments and positive relationships with clinical staff for patient well-being, factors which should not be neglected in the planning and delivery of health and social care services for patients with advanced illness.

## Abbreviations

CG, control group; IMP, investigational medicinal product; IG, intervention group; I, interviewer; IPA, Interpretative Phenomenological Analysis; P, participant
